# Assessment of Potential and Techno-Economic Performance of Solid Sorbent Direct Air Capture with CO_2_ Storage in Europe

**DOI:** 10.1021/acs.est.3c10041

**Published:** 2024-06-03

**Authors:** Tom Terlouw, Daniel Pokras, Viola Becattini, Marco Mazzotti

**Affiliations:** † Separation Processes Laboratory, Institute of Energy and Process Engineering, ETH Zurich, Zurich 8092, Switzerland; ‡ Chair of Energy Systems Analysis, Institute of Energy and Process Engineering, ETH Zurich, Zurich 8092, Switzerland; § Technology Assessment Group, Laboratory for Energy Systems Analysis, 5232 Villigen PSI, Switzerland

**Keywords:** techno-economic assessment, direct air capture with CO_2_ storage (DACCS), carbon dioxide removal (CDR), negative emission technologies (NETs)

## Abstract

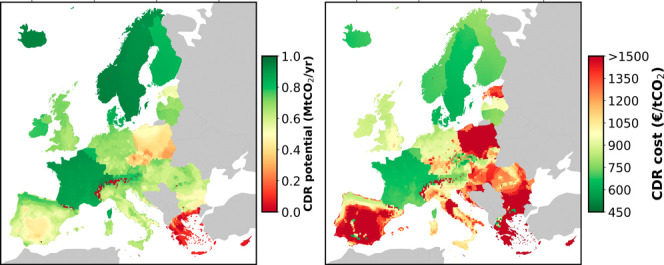

Direct air capture with CO_2_ storage (DACCS) is among the carbon dioxide removal (CDR) options, with the largest gap between current deployment and needed upscaling. Here, we present a geospatial analysis of the techno-economic performance of large-scale DACCS deployment in Europe using two performance indicators: CDR costs and potential. Different low-temperature heat DACCS configurations are considered, i.e., coupled to the national power grid, using waste heat and powered by curtailed electricity. Our findings reveal that the CDR potential and costs of DACCS systems are mainly driven by (i) the availability of energy sources, (ii) the location-specific climate conditions, (iii) the price and GHG intensity of electricity, and (iv) the CO_2_ transport distance to the nearest CO_2_ storage location. The results further highlight the following key findings: (i) the limited availability of waste heat, with only Sweden potentially compensating nearly 10% of national emissions through CDR, and (ii) the need for considering transport and storage of CO_2_ in a comprehensive techno-economic assessment of DACCS. Finally, our geospatial analysis reveals substantial differences between regions due to location-specific conditions, i.e., useful information elements and consistent insights that will contribute to assessment and feasibility studies toward effective DACCS implementation.

## Abstract 2

We provide a geospatial analysis to quantify the potential and techno-economic performance of different grid-connected direct air capture with CO_2_ storage configurations toward large-scale deployment in Europe.

## 1 Introduction

Limiting global warming to 1.5 °C implies reaching net-zero CO_2_ emissions globally by around 2050.^1,2^ To achieve this target, all anthropogenic emissions must be minimized, and any remaining hard-to-abate emissions should be balanced by an equivalent amount of carbon dioxide removal (CDR) from the atmosphere. CDR approaches can deliver such net-negative CO_2_ emissions, which should be achieved and sustained in the second half of the century to reverse the increase in global mean surface temperature.^1,2^

Among the CDR options, direct air capture with CO_2_ storage (DACCS) is still a relatively novel technology (technology readiness level of 7)^3^ with a significant projected future CDR demand estimated between 5 and 40 Gt CO_2_/year.^1,4−6^ This CDR approach relies on the combination of two technologies: removal of CO_2_ from the atmosphere through chemical processes and subsequent permanent CO_2_ storage, generally accomplished by injecting CO_2_ in a geological formation underground.^7,8^

Besides the growing literature on DACCS,^7−17^ commercial firms are currently piloting this technology. Carbon Engineering (Canada) has been developing an aqueous direct air capture (DAC) system based on Ca-looping technology,^14^ while Climeworks (Switzerland)^18^ has been developing DAC units based on amine-functionalized adsorbents. In addition to these firms, which have been pioneering DAC in the past decade, other private initiatives are developing alternative approaches based on different materials and at different scales.^19−21^ For example, Verdox uses electric swing adsorption without the need for low-temperature heat requirements and claims higher process efficiencies.^22^ Instead, Noya’s technology uses activated carbon monoliths coated with CO_2_ absorbing chemicals enabling the separation of CO_2_ from the atmosphere.^19^ Here, our focus is on solid sorbent low-temperature DAC solutions. One of the advantages of low-temperature DAC is their modularity and low-temperature heat requirements during regeneration as opposed to solvent-based high-temperature DAC systems, which are subject to high-temperature heat demand.^23^

Regardless of the capture method and scale, all DAC technologies exhibit considerable energy requirements due to the extreme dilute CO_2_ concentration in atmospheric air. DAC should, therefore, be powered by decarbonized energy sources to ensure a high CDR efficiency.^7,8^ Further, as DAC is a relatively new technology, it is associated with high capital investments although learning by doing and large-scale deployment are supposed to substantially lower total CDR costs.^12,23^

After capture, the CO_2_ must be transported to a dedicated site for permanent storage in an underground geological formation. In Europe, CO_2_ storage hubs are currently being developed in the North Sea, thus, could provide CO_2_ storage for DAC. In fact, DAC is a flexible technology and not linked to a specific point source, and hence can be placed next to a storage site in order to minimize CO_2_ transport and associated environmental and economic burdens. For example, the largest DAC plant—with an installed capacity of 36,000 t CO_2_/y using Climeworks’ technology—has been set into operation in May 2024 in Iceland,^24^ where the CO_2_ is injected underground in a basalt formation for permanent storage through mineralization by the Icelandic company Carbfix.^25^ Iceland provides exceptionally ideal conditions for DACCS, offering both abundant low-carbon geothermal energy to power DAC and suitable geological conditions to allow for permanent CO_2_ storage, thus eliminating the need to transport CO_2_ over long distances between capture and storage sites. However, Iceland is a unique case; large-scale deployment of DACCS will imply finding a trade-off between the proximity of clean energy sources and geological storage sites. Should one of these conditions not be met, DACCS will likely rely on locally available energy sources with different greenhouse gas (GHG) emission factors, depending on their location and long-distance CO_2_ transport to a geological storage formation.

A few recent studies have investigated the effects of location-specific factors on the techno-economic performance of DAC, and possibly on CO_2_ storage, in terms of energy consumption and costs. Sendi et al. analyzed the effect of climate on DAC performance, providing a regional economic assessment of sorbent-based DAC systems.^26^ The authors identified the most suitable climates and regions for DAC deployment using a spatial resolution of 0.5° × 0.625°, finding that colder and drier regions are the most suitable for DAC—and the selected sorbent—when the cost of capital is not taken into account. Wiegner et al. investigated the performance of a reference sorbent-based DAC process under varying ambient conditions, i.e., temperature and humidity, and computed the energy consumption and the minimum system costs for multiple temperature–humidity combinations and three exemplary locations.^27^ The optimal performance of the system can be achieved by adjusting the operating conditions based on the ambient air conditions (i.e., generic climate conditions) and when flexibility in CO_2_ production can be guaranteed, e.g., through a buffer storage tank. Young et al. calculated the DACCS costs for four example technologies, and for plants that may be built both today or in the future.^28^ The authors indicate that the costs of the first plants will be higher than many figures quoted today, and argue that in the long term, costs may decrease to 80–600$ t CO_2_^–1^ at the Gt CO_2_/y scale. Further, proper siting and energy source selection are critical to minimize DACCS costs.

Although these studies provide valuable insights for the selection of suitable locations for DAC and the assessment of its efficiency and costs, we find that CO_2_ transport and its associated environmental and economic costs are usually neglected, arguably unreasonably, as their contribution can be significant,^8^ especially considering that DAC units may be placed at large distances from storage sites.^29^ Further, prior analyses have neglected (i) location-specific waste heat potential and typically (ii) the influence of the local climate on the performance of grid-connected DACCS, except for some of the studies mentioned in the previous paragraph. Here, we aim to close these research gaps by determining the optimal geospatial distribution of potential grid-connected DAC plants in Europe concerning the availability of energy sources and the vicinity of geological formations for permanent CO_2_ storage underground. Different from previous works, our work provides the following novelties and key contributions:

- Suitable and less-suitable locations are identified for grid-connected DACCS in a European context using a fine spatial resolution (0.25° × 0.25°). Such resolution is especially important to characterize the performance of the solid sorbent and the energy demand for CO_2_ capture, which are climate dependent. Different DAC configurations are considered based on locally available energy sources, such as electricity from the power grid, heat from waste heat sources, or heat produced with a high-temperature heat pump;
- European waste heat availability and renewable energy curtailment are determined, which can be used to provide low-temperature heat to DACCS facilities. The assessment of these energy sources allows to determine the DACCS potential when relying on such sources for CO_2_ capture. In addition, country-specific CDR potentials based on grid-connected DACCS are provided, and a critical reflection on the availability of waste heat in the future is given in the discussion;
- The role and impact of CO_2_ transport and storage, and of different clean energy supplies for grid-connected DACCS are determined.

Section 2 presents the scope and methodology, including the evaluation metrics used. After that, the results and discussion are presented in Sections 3 and 3.5, respectively. Finally, the implications of grid-connected DACCS deployment are drawn in Section 4.

## 2 Scope and Methodology

The geographical scope of the work covers the EU/EEA member states, which are seeing the establishment and development of several pioneering initiatives directed toward the large-scale deployments of DAC, underground CO_2_ storage, and CDR technologies.^18,25^ Furthermore, the European Union foresees DACCS playing a key role among CDR methods to achieve climate neutrality, as it emerges from the targets set in the recent Climate Law.^30,31^

The DACCS supply chains considered and modeled here consist of a DAC unit, a CO_2_ transport, and a CO_2_ storage. The potential of these supply chains is assessed in terms of CDR potential (i.e., Mt CO_2_/year) and costs (i.e., net costs per tonne of CO_2_ removed, € t CO_2_^–1^). Next, we describe how various steps of the DACCS supply chains are modeled.

### 2.1 DAC Unit

The DAC unit considered in this study is based on a temperature-vacuum swing adsorption (TVSA) process using a porous sorbent material with a high specific surface area, similar to the sorbent used by the Swiss DAC company Climeworks.^18,32,33^ The TVSA is a cyclic process that consists of four main steps: (i) adsorption of CO_2_ from the atmosphere, (ii) preheating and depressurization toward vacuum pressures to evacuate the contactor and to remove other gases, (iii) heating under vacuum and desorption of CO_2_, and (iv) cooling and repressurization. Other variations include injecting steam under a vacuum to lower the partial pressure of CO_2_ and to extract larger amounts of CO_2_ during the desorption step.

The energy consumption of the TVSA process is highly sensitive to air inlet conditions (e.g., temperature and relative humidity) and has been recently modeled (both electricity and heat requirements) by Wiegner et al.^27^ In our geospatial analysis, we utilize the correlations provided by those researchers^27^ to estimate the heat and electricity consumption of the process as a function of the climate of a specific geographical region.

**Figure 1:**
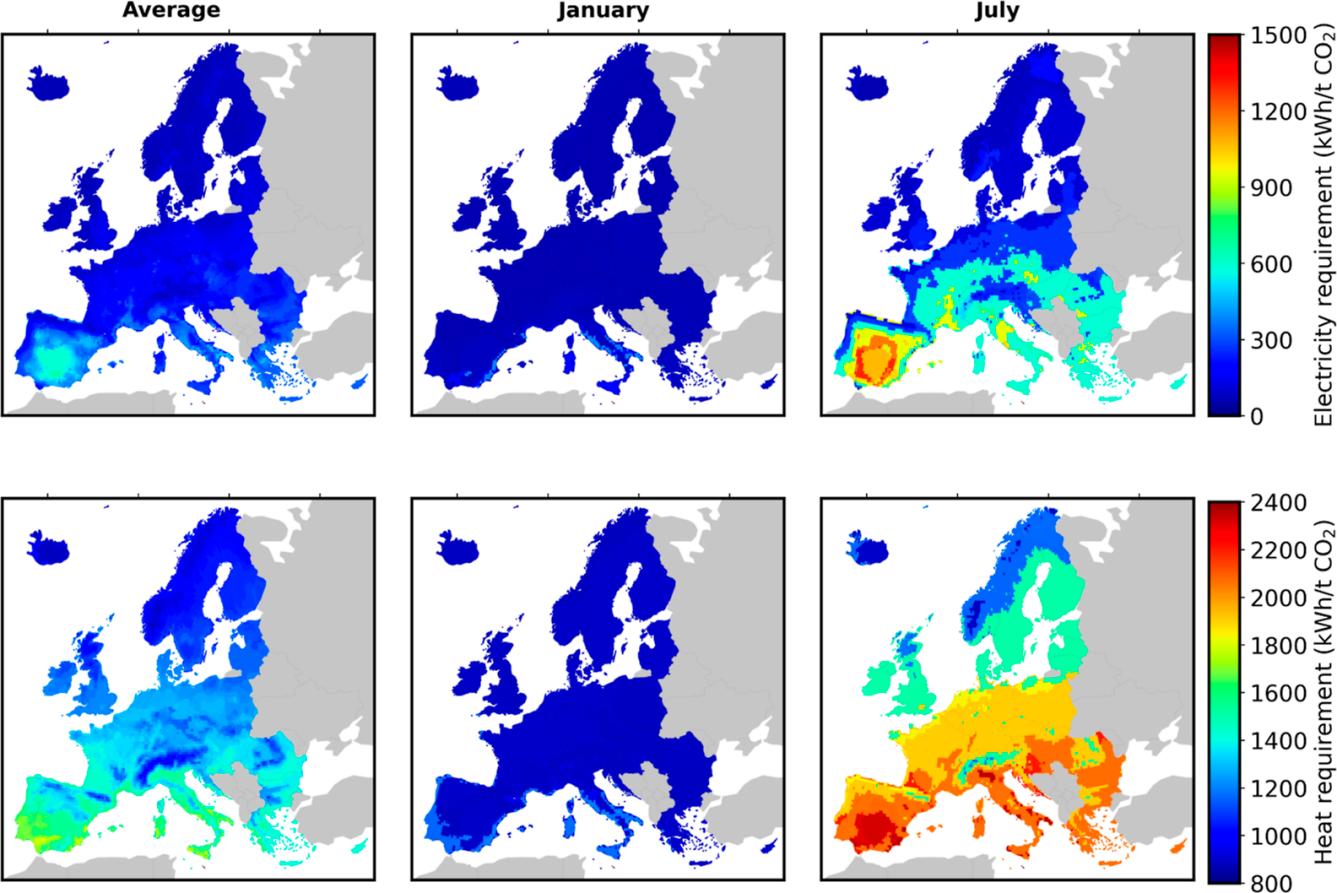
Location-specific energy demand (in kWh tCO_2_^–1^) computed for DAC for three scenarios, i.e., (a) Average, (b) January, and (c) July.

The spatial resolution of our geospatial analysis is 0.25° × 0.25°, which corresponds (depending on latitude) to approximately 27.8 × 20 km in Europe. The location-specific temperature (rounded to the nearest multiple of 5 °C) and the relative humidity values (rounded to the nearest multiple of 10%) are determined through satellite data for Europe,^34^ collected with a resolution of 0.25° × 0.25° over a ten-year period (i.e., from 2011 to 2021). The data are averaged to create a representative climate database for each location (i.e., cell) and each month based on the last ten years. Additionally, for relative humidity, the average monthly values of nearby cells are used for locations where climate data are missing (for about 20% of the cells, mostly corresponding to Greece, Bulgaria, and Cyprus). For each cell, the monthly averaged and location-specific DAC performance (i.e., specific energy consumption) is calculated using the model provided by Wiegner et al.^27^Figure 1 illustrates the location-specific energy demand computed for DAC for the annual average and for two specific months as well, i.e., January and July. The monthly total energy requirements for DAC are illustrated in Figure S2 of the Supporting Information.

#### 2.1.1 Energy Supply Scenarios

Previous analyses on DAC demonstrated that energy supply is one of the key factors determining its economic and environmental potential.^7,8^ Here, we consider various DAC configurations relying on different energy supply scenarios for CO_2_ capture, in terms of both electricity and heat supply. More specifically, three different energy supply scenarios are investigated:

(1) DAC powered by electricity from the grid, and heat supplied by a high-temperature heat pump (HTHP) operated with electricity from the grid.
(2) DAC powered by electricity from the grid and heat supplied locally by available waste-heat sources.
(3) DAC powered by electricity from curtailed renewable sources, and heat supplied by a HTHP operated with curtailed renewable electricity.

Further explanations of these scenarios are given, together with the results in Section 3. An overview table with the main assumptions of the scenarios is given in Table 1. It is worth noting that locations with elevations higher than 2000 m altitude have been removed from our analysis due to lower air density and CO_2_ partial pressure, its influence on DAC performance, and the difficulty of transporting energy and infrastructure to such geographical locations.

**Table 1:** Assumptions Used in the Different Scenarios

DAC scenario	unit	grid power + heat from HTHP	grid power + waste heat	curtailed power + heat from HTHP
energy sources	[−]	grid power	grid power and waste heat	curtailed power from renewables
electricity price	[€ kWh^–1^]	0.042–0.133	0.042–0.133	0
heat price	[€ kWh^–1^]	depends on CoP and power price	0	depends on CoP and power price
GHG-intensity	[kg CO_2_-equiv kWh^–1^]	0.026–1.035	0.026–1.035	0
HTHP–CoP	[−]	2.9	2.9	2.9
max. DAC size	[Mt/a]	1	12 (depends on local waste heat)	5 (depends on renewable plant)

### 2.2 CO_2_ Transport

After being captured, the CO_2_ is transported to a geological storage site, see Section 2.3. Currently, there exists no cross-border infrastructure to transport CO_2_. For simplicity and due to substantial uncertainties with regard to the transport of CO_2_, we have considered the unitary costs (€ t CO_2_^–1^/km) of pipeline transportation using 0.04 € t CO_2_^–1^/km and 32 gCO_2_-equiv tCO_2_^–1^/km.^35^

The distance for any given DAC facility to the nearest geological storage location is based on the most direct path, i.e., the straight-line distance. In other words, captured CO_2_ is always assumed to be delivered to the nearest storage site, regardless of its capacity.

### 2.3 CO_2_ Storage

Underground geological storage of CO_2_ has been gaining traction in Europe in recent years. Several initiatives and projects are currently in development for CO_2_ storage in the region, especially in the North Sea, expected to be operational by 2024 onward and often promoted by consortia of European energy companies. Nevertheless, there still exists a high level of uncertainty related to the maximum capacities of storage sites under development and to the future establishment of new sites, which will continue to be announced. Therefore, there is general consensus that the CO_2_ storage landscape will change significantly in the coming years. For these reasons, we decided here to consider potential storage sites in saline aquifers in Europe rather than relying only on announced commercial storage initiatives.^36^ In addition, this assumption will shed light on where it would be most effective to develop new CO_2_ storage sites to serve DAC facilities. The considered geological storage locations are also presented in Figures 5 and 6 (star symbols). In this context, we focus on theoretical storage locations having storage capacities larger than 5 Gt of CO_2_ since we are interested in the large-scale deployment of DACCS. Further, we exclude existing storage projects designated for regional industries planning to use carbon capture and storage (CCS). The cost of geological storage is assumed to be 5–27 € t CO_2_^–1^ with an applied median value of 11 € t CO_2_^–1^.^28^ Further techno-economic data used are given in Table 2.

**Table 2:** Techno-Economic Data Used

variable	value	unit	ref
cost of storage	11	€ t CO_2_^–1^	(28)
fuel cost–electricity	variable (see Table S1)	€ kWh^–1^	(37)
interest Rate (WACC)	variable (see Table S1)	%	(38), (39)
GHG intensity electrical grid	variable (see Figure 2a)	kg CO_2_-equiv kWh^–1^	(40)
DAC reference capex	6.94	million €	(28)
DAC opex	4% of capex	million €	(12)
DAC adsorbent costs	37	€ t CO_2_^–1^	(28)
DAC reference capture capacity	960	tCO_2_ year^–1^	(28)
DAC plant emissions	6	kg CO_2_-equiv t CO_2_^–1^	(7)
DAC adsorbent production emissions	24	kg CO_2_-equiv t CO_2_^–1^	(7)
heat pump capex	600	€ kW^–1^	(41)

### 2.4 Evaluation Metrics

The viability of DACCS in a given region is assessed based on two key performance indicators (KPIs): the net CDR potential (*m*_CDR_, Mt CO_2_/y) and net CDR cost (*C*_CDR_, € t CO_2_^–1^). The CDR potential corresponds to the yearly net CO_2_ removed from the atmosphere after accounting for gray emissions along the entire supply chain (e.g., to power DAC). The CDR cost corresponds to the yearly total cost divided by the total net CDR potential. While the former is calculated for all three energy supply scenarios considered, the latter is calculated only for the first two scenarios (based on grid electricity) due to the low CDR potential estimated from DAC powered by curtailed renewable electricity.

The CDR potential is calculated for a given location where DAC could be installed and assumes full utilization of all available energy resources without competing with other potential nearby locations. As explained in Section 2.1, the gross DAC potential (*m*_DAC_, Mt CO_2_/y) is calculated based on the DAC performance model presented elsewhere,^27^ i.e., considering the available heat and electricity, and the specific energy consumption of DAC at a given location. The CDR potential is then computed as the mass of CO_2_ captured by the DAC unit minus the GHG emissions caused within the whole DACCS supply chain, grouped in the term *m*_losses_ in eq 1
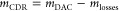
1

The GHG emissions considered are those incurred from capturing, transporting, and storing the CO_2_. Thus, we include GHG emissions from the construction and operation of the DAC unit itself, sorbent material, waste-heat losses, absorbing electricity from the power grid, and direct and indirect emissions of CO_2_ transport and storage. The CDR cost is calculated based on the costs incurred throughout each step of the DACCS supply chain
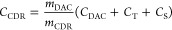
2where *C*_DAC_, *C*_T_, and *C*_S_ are the levelized costs of capture, transport, and storage, respectively (in € t CO_2_^–1^). The levelized cost of DAC comprises capex, fixed operational expenditures (opex), and fuel expenditures, i.e., electricity and heat
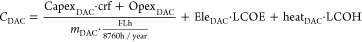
3where an annualization period of 20 years (*N* = 20), full load hours (FLh) are assumed to be 8000 h, and a location-specific cost recovery factor (crf) based on the country-specific weighted cost of capital (WACC) are assumed
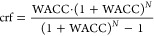
4

The DAC opex are assumed to be 4% of the capex,^12^ while the DAC capex costs (Capex_DAC_) are scaled depending on the DAC plant size (*S*_DAC_) using the power law equation
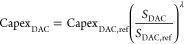
5where the reference capex (Capex_DAC,ref_) is derived from Young et al.^28^ and is based on estimates for the Climeworks DAC plant in Hinwil, Switzerland, having a gross capture capacity of 960 t CO_2_/y (i.e., size, *S*_DAC,ref_) and a capex of ca. 7 M€. The scaling factor λ is estimated to be 0.91. Given the modular nature of the DAC technology, this factor is based on the mass production rule, which estimates a linear increase in material needed as a function of the installed capacity plus a 10% margin considering improvements of other components of the DAC infrastructure. This choice can be regarded as conservative (especially for large plant sizes) as, in practice, units such as heat exchangers and pipe connections within the plant typically have much lower cost scaling factors.^42^ Thus, we apply a conservative scaling factor in the main analysis and provide additional sensitivity analyses by applying a set of other scaling factors in Figures S4 and S5 of the Supporting Information.

The energy expenditures are calculated based on the location-specific DAC electricity and heat consumption per ton of CO_2_ captured (Ele_DAC_ and heat_DAC_, respectively, in kWh t CO_2_^–1^), and on the levelized costs of electricity and heat (LCOE and LCOH, respectively, in €/kWh), which depend on the energy supply scenario considered.

The LCOE is based on the country-specific grid electricity price: scenarios (1) and (2). Under energy supply scenario (1), the LCOH is calculated based on the HTHP capex and opex as well as the electricity costs to operate it. For scenario (2), the LCOH is a function of the cost of the infrastructure (i.e., the pipe) needed to connect the waste heat source to the DAC site
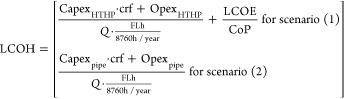
6with Capex_pipe_ and Opex_pipe_ being the capital and operational costs for the pipe for transporting heat, and *Q* the yearly installed capacity of the pipe (i.e., kWh_th_/y). These terms are calculated based on a cost optimization developed by ref (43). The levelized CO_2_ transport costs (*C*_T_) are calculated based on the transport unitary costs using pipelines (see the cost figures in Section 2.2) and the distance between the DAC plant and the nearest storage facility.

## 3 Results and Discussion

The presentation of the results is structured according to the energy supply scenario considered (see Section 2.1.1). First, the results of (1) DAC powered by electricity from the grid and (2) DAC powered by electricity from the grid and heat supplied locally by available waste-heat sources are presented in Sections 3.1 and 3.2, respectively. Second, the results of DAC powered by electricity from curtailed renewable sources are provided in Section 3.3. Third, the results of the sensitivity analysis are provided in a Section 3.4. Finally, further discussions are given in Section 3.5.

### 3.1 DAC Powered by Grid Electricity and HTHP

**Figure 2:**
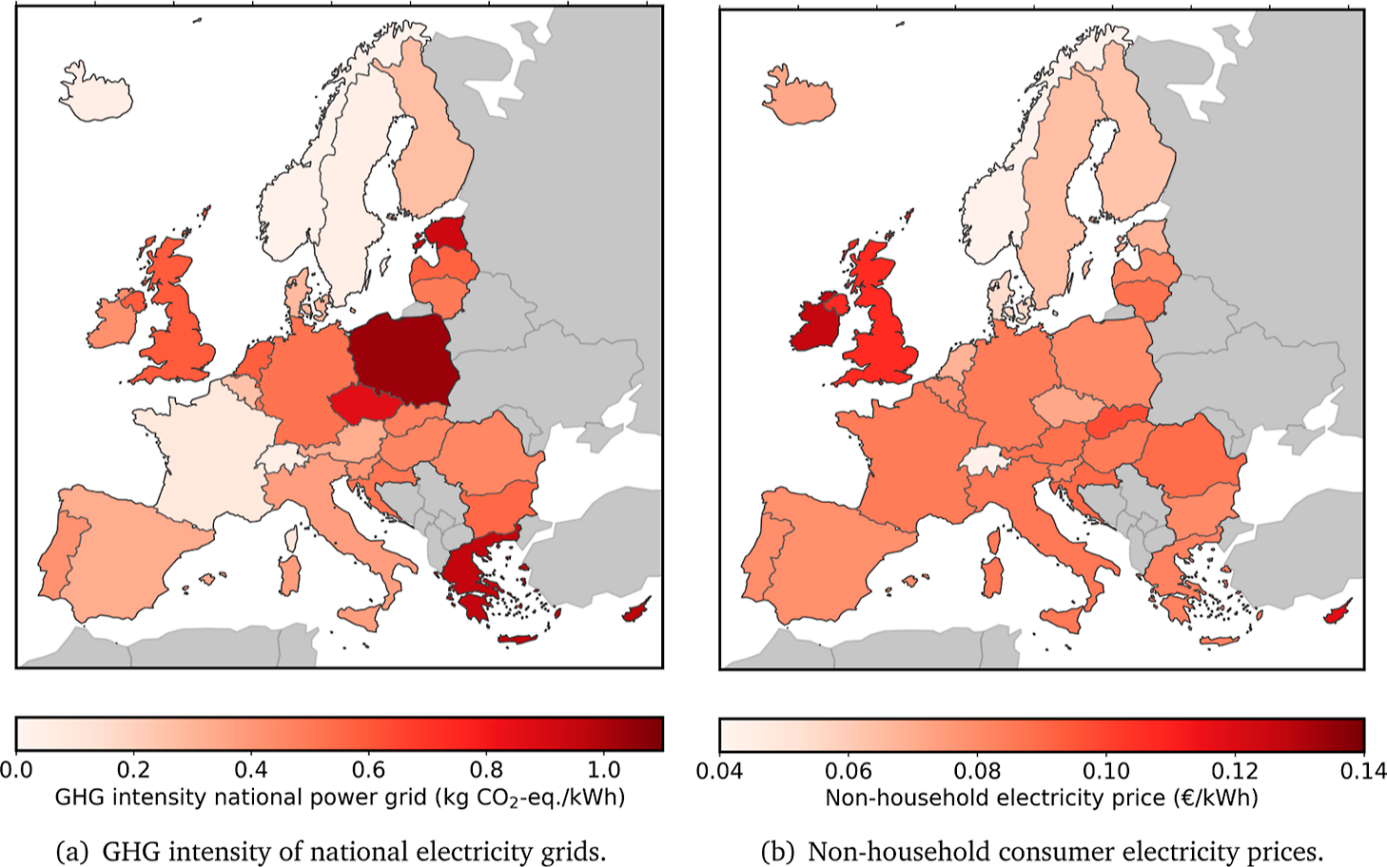
Data on national power grid GHG intensity to the left, nonhousehold consumer electricity prices to the right.

In this scenario, the electricity source is assumed to be virtually available anywhere throughout Europe and accessible via the local grid electricity network. Sensible heat, required for the regeneration step, is produced using an HTHP with a coefficient of performance (CoP) of 2.9.^8^ The price and GHG intensity of the grid electricity depend on the local grid electricity network and is country-specific. Electricity prices are obtained from the biannual nonhousehold consumer electricity prices from the Eurostat database, see Figure 2b.^37^ GHG emission factors from grid electricity absorption, see Figure 2a, are obtained from the ecoinvent database (v3.8) using the system model “Allocation, cutoff by classification”.^40,45^ Given the lack of a quantifiable upper limit for the energy input in this scenario, each potential DAC unit is assumed to have a maximum annual CO_2_ capture capacity of 1 MtCO_2_ corresponding to the capacity of two large-scale DAC plant currently built in the USA.^46^ This upper limit is applied to each grid cell to reflect potential constraints that are likely limiting the availability of grid electricity for CO_2_ capture, such as (expensive and unfeasible) grid network expansion requirements, social acceptance, and land availability and competition.

**Figure 3:**
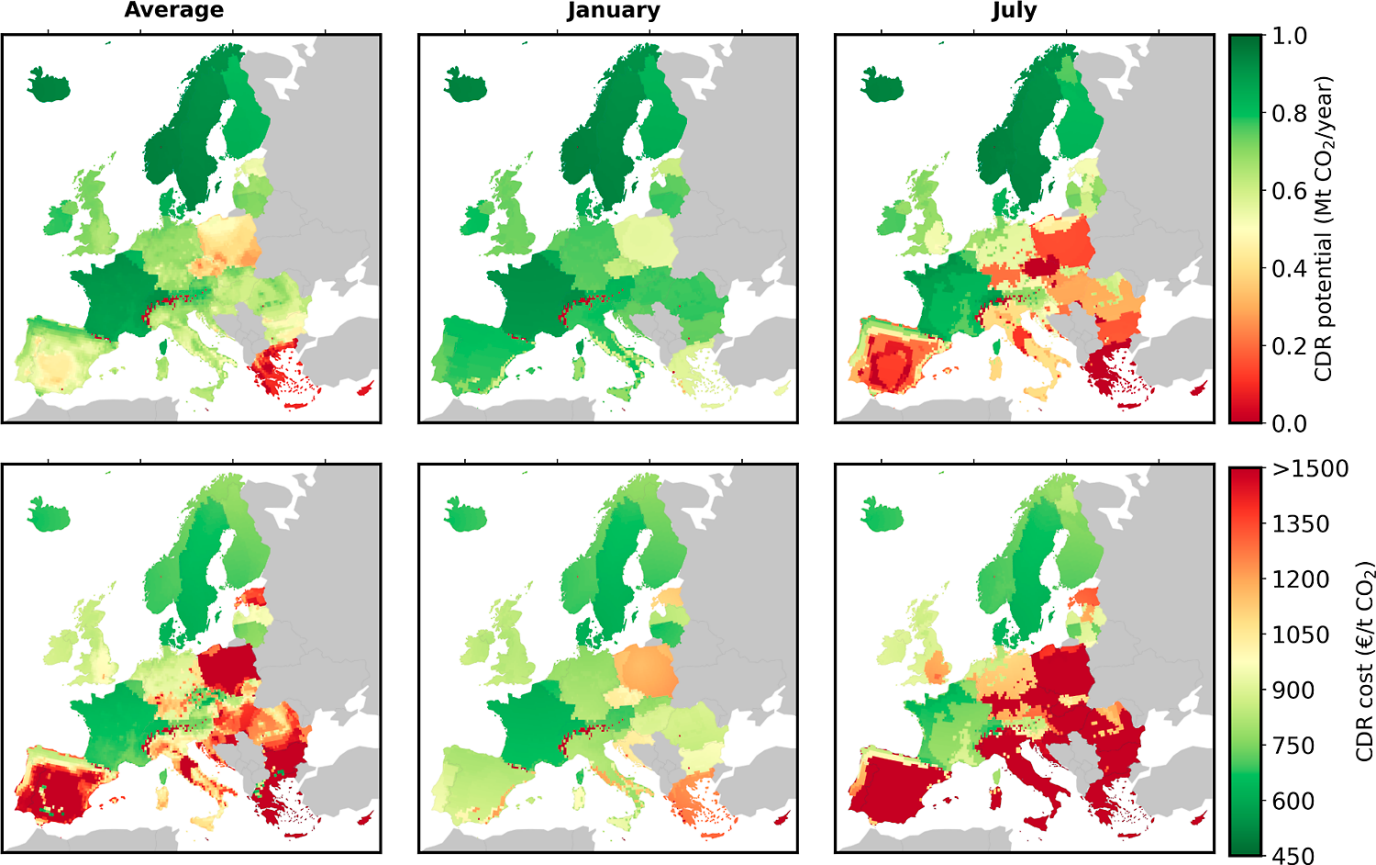
DACCS supply chains relying on DAC powered by grid electricity and HTHP; CDR potential (Mt CO_2_/y) on the first row and CDR costs (€ t CO_2_^–1^) on the second row. The six subplots illustrate different average ambient air conditions (temperature and relative humidity) on the columns; annual average, January, and July. The results for all months are provided in Figure S3 of the Supporting Information.

Figure 3 shows six maps with European geographical scope. The three subplots in the first row of Figure 3 illustrate the CDR potential (in Mt CO_2_/year) that can be realized through DACCS in Europe if grid electricity and high-temperature heat pumps are deployed as energy sources for CO_2_ capture. The maximum attainable potential is constrained to one Mt of CO_2_/year. In other words, the maps show the regions that are most attractive to implement DACCS with respect to CDR efficiency (dark-green areas, e.g., France, Iceland, Sweden, Switzerland, and Norway), compared to regions that are less suitable or not suitable (dark-red areas, e.g., Greece, Poland, and the Czech Republic). Three scenarios are included to demonstrate the influence of ambient air conditions on energy requirements for CO_2_ capture; temporal average (average annual relative humidity and temperature), average for a winter month (January), and average for a summer month (July). The color bar quantifies the CDR efficiency, ranging from “0” or lower (in dark red, no CDR) up to “1” (in dark green, a CDR efficiency of 100%).

In addition, the three subplots on the second row of Figure 3 show the location-specific CDR costs (in € t CO_2_^–1^) for the European locations considered. Similarly to the first row of Figure 3, three scenarios (average, January, and July) are considered to illustrate the influence of ambient air conditions on the CDR costs. The color bar quantifies the CDR costs ranging from 450 € t CO_2_^–1^ or lower (dark green colored) up to 1500 € t CO_2_^–1^ or higher (dark red colored). It should be noted that these costs are representative of a young technology, such as DAC, which still has a low technology readiness level. In the future, cost reductions, due to e.g., learning-by-doing, are expected to decrease.^28,47^ Therefore, concerning the data presented in this study, we encourage readers to consider them in a relative context, focusing on comparative trends rather than on absolute values.

Under this scenario, the CDR potential and costs are mainly driven by the interplay of different factors: (i) the conditions of ambient air, (ii) the LCOE and the GHG intensity of the electricity consumed, and (iii) the distance to a CO_2_ storage site and the associated CO_2_ transport costs. As the energy for CO_2_ capture is entirely provided by the national power grid, countries with a GHG-intensive power grid incur larger CDR costs. In addition, the cost grows with increasing distance between the DAC location and the storage site, i.e., when the CO_2_ transport distance and costs increase, thus penalizing southern countries located at a thousand(s) kilometers distance from geological storage sites in northern Europe.

Importantly, ambient air conditions are a crucial factor for CDR efficiency and costs since they significantly influence the capture efficiency of the DAC plant and its energy consumption; see Figure 1. More specifically, for the selected sorbent, mountain and coastal regions have on average higher CO_2_ capture efficiencies due to more beneficial climate conditions, such as a higher average relative humidity and a lower ambient temperature. Further, the seasonal influences are significant; this is visible through the substantial differences when applying the ambient air conditions of the annual average, of a typical winter month (January), and of a typical summer month (July). This seasonal effect is especially noticeable in geographical locations with substantial ambient air differences between the winter and summer months. For example, in Spain, there are geographical regions with average monthly CDR costs of approximately 800 € t CO_2_^–1^ during winter, while the CDR costs reach up to 1500 € t CO_2_^–1^ (or more) in summer months. Geographical regions that exhibit robust CDR potential throughout the entire year (higher than 0.75 Mt CO_2_/y) and costs (below 700 € t CO_2_^–1^) are mainly situated in the North of Europe.

Overall, we find that CDR potential and cost are the highest and the lowest, respectively, in geographical areas (i) with the availability of a decarbonized national power grid, (ii) in the vicinity of a geological CO_2_ storage site, and (iii) with suitable climate conditions (i.e., a cold and humid climate for the selected sorbent). Such suitable geographical locations can, for example, be found in Denmark, Switzerland, the Northwest of France, Sweden, and Iceland, which aligns with recently installed solid sorbent-based DAC facilities of Climeworks in Hinwil (Switzerland) and Hellisheidi (Iceland).

#### 3.1.1 Contribution Analysis

**Figure 4:**
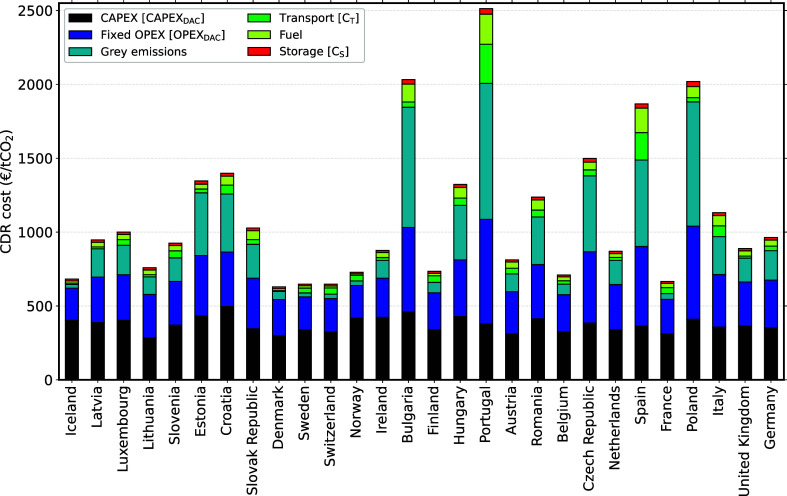
Contribution analysis of DAC powered by grid electricity and HTHP. The values are averaged for each country considered and represent the average annual climate conditions.

Figure 4 illustrates the main contributions to the CDR costs, such as capital (CAPEX), fixed operational expenditures (Fixed OPEX), fuel costs, transport of CO_2_, storage of CO_2_, and gray emissions from fuel (e.g., electricity), construction, transport, and storage of CO_2_. These cost contributions are illustrated in different colors. The list of European countries considered is on the *x*-axis, while their national average CDR costs are on the *y*-axis.

Major cost contributions are due to capital, fixed OPEX, and gray emissions for countries with a GHG-intensive power grid. The fuel and storage costs make a rather small contribution to the overall CDR cost. CO_2_ transport costs can be significant for locations without the proximity of a CO_2_ storage formation, for example, in Spain and Portugal.

**Figure 5:**
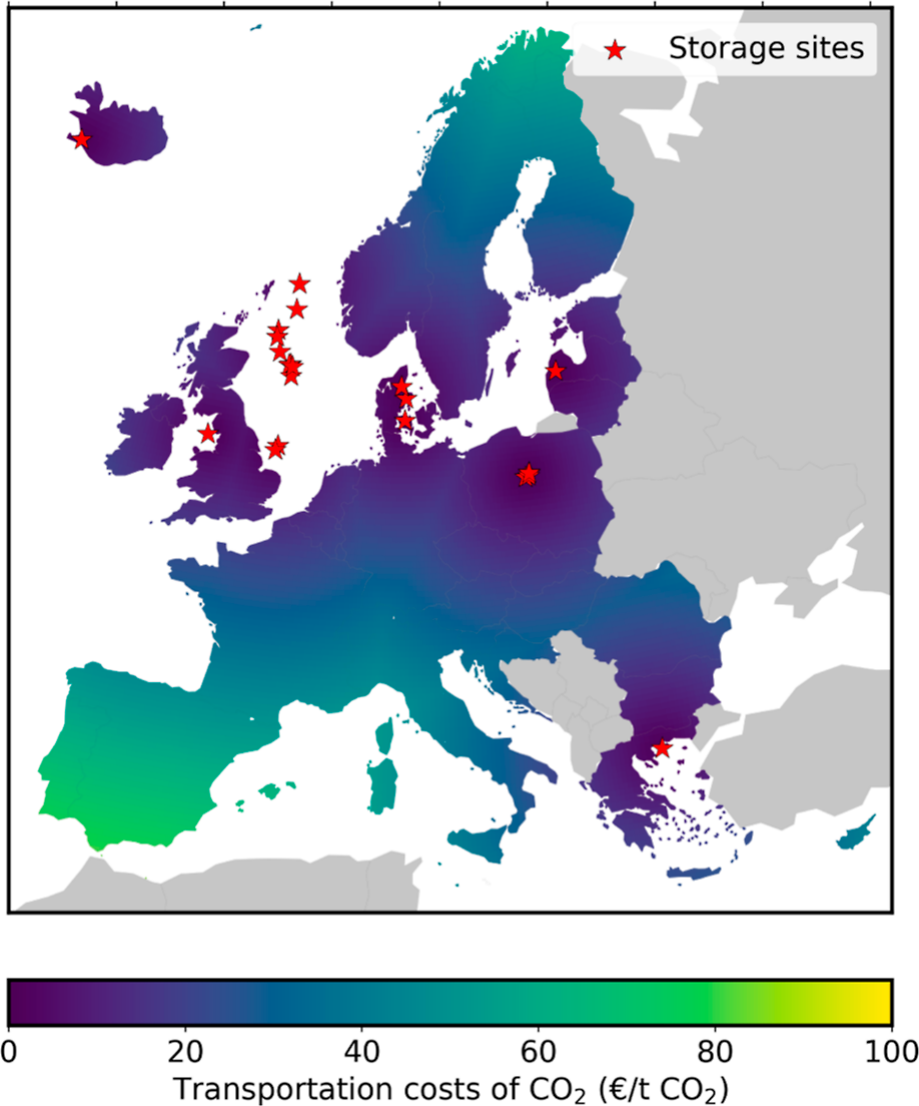
Total transport costs of CO_2_ (€ t CO_2_^–1^) from a given European location to the closest CO_2_ geological storage site assumed.

Figure 5 illustrates that the costs of CO_2_ transport can be an important contributor to the CDR cost when the distance between the DAC plant and the nearest geological storage site is large (more than 1500 km); such costs could have a share of up to 10% of the total cost in, for example, Portugal or Spain, even when assuming a large-scale, low-cost CO_2_ transport mode as pipelines. Such transport costs would increase significantly if pipelines were not available and other discontinuous batch-based transport modes were deployed (e.g., trains or trucks).^35^ Overall, the transport component must be considered in a comprehensive assessment of more and less suitable geographical locations for the large-scale deployment and roll-out of DACCS.

### 3.2 Waste Heat Sources with Grid Electricity

**Figure 6:**
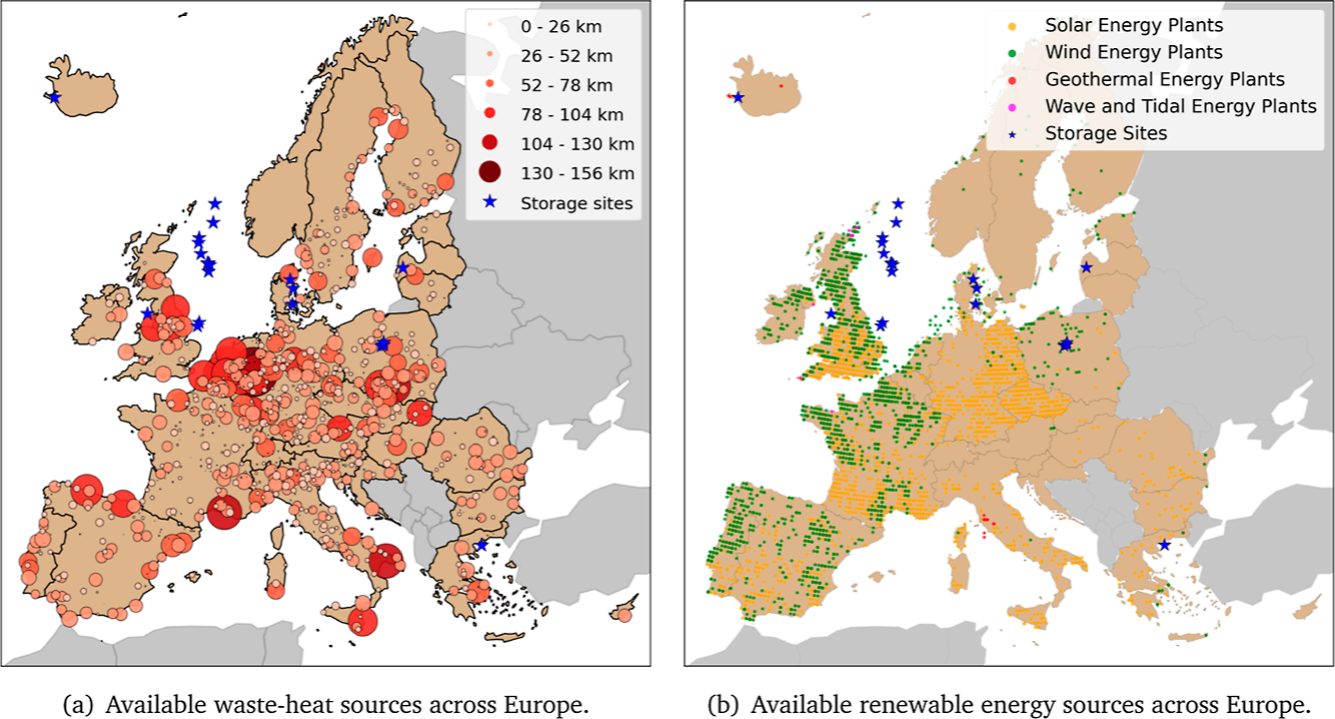
Data on available waste heat sources to the left, and renewable energy generation plants to the right.

In this scenario, heat for powering DAC is provided by industrial waste-heat sources within Europe (e.g., from pulp and paper, nonmetallic minerals, iron and steel, refineries, and chemical industries). Similar to the previous scenario, the electricity price and GHG intensity depend on the local power grid. The location-specific waste-heat potential is assessed based on available data and is represented in Figure 6a.^48^ The data were filtered to use only heat sources at temperatures larger than 95 °C (see Figure 6a), which have been aggregated for heat sources that are within a 10 km radius of each other. The color of the bubbles (different red colors) illustrates the scale combined with the amount of waste heat available, while their size represents the maximum feasible distance of heat transport using water as the transport fluid, given the quantity of waste heat available. Overall, the waste-heat potential that could be deployed for DAC in Europe has been estimated to be about 427 PJ.

**Figure 7:**
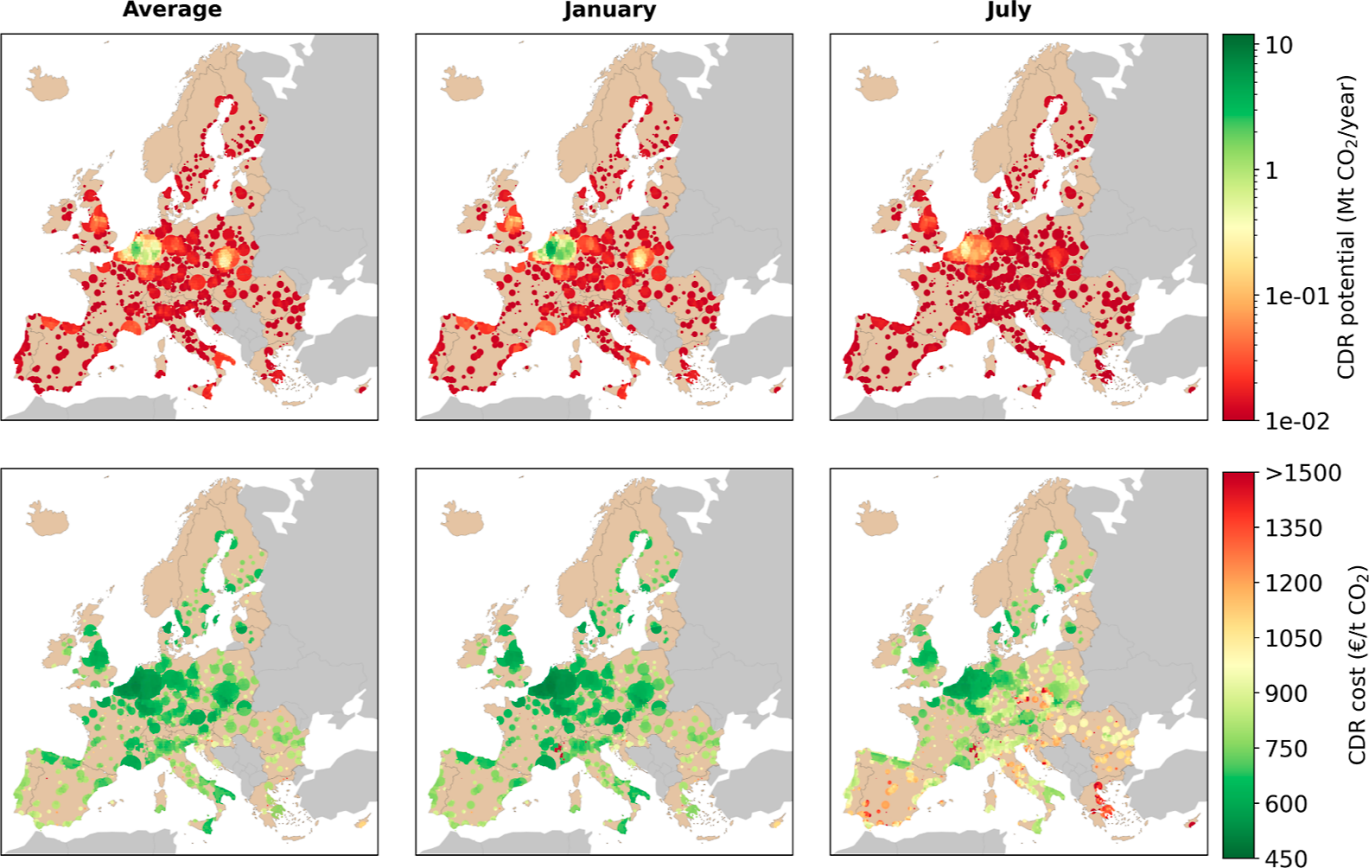
DACCS supply chains relying on DAC powered by waste heat and grid power; CDR potential (Mt CO_2_/y) on the first row and CDR costs (€ t CO_2_^–1^) on the second tow. The six subplots illustrate different average ambient air conditions (temperature and relative humidity) in the columns; annual average, January, and July.

**Figure 8:**
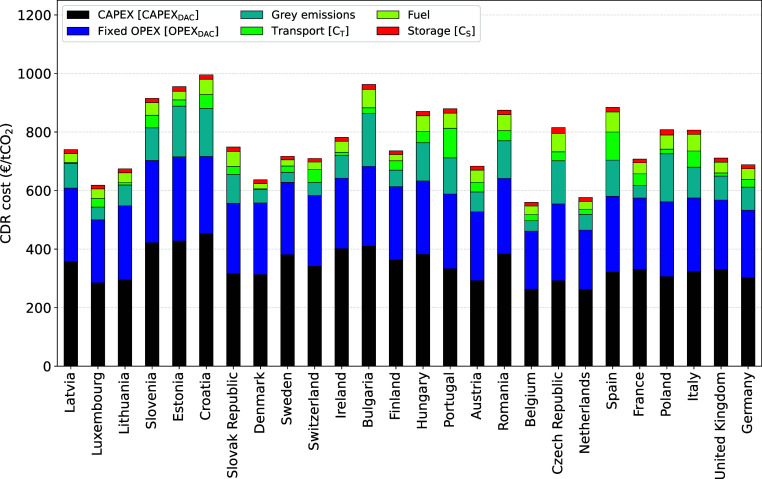
A bar plot illustrating a contribution analysis of the national averaged CDR costs for the European countries considered for the waste heat alternative (on the *x*-axis). The *y*-axis shows the total CDR costs up to 1200 euro/t CO_2_ removed. The size and colors of the bar segments represent the contributions of specific processes to the total net costs of CO_2_ removal.

Figure 7 illustrates the CDR potential and cost achieved through DACCS using grid electricity and industrial waste heat, while a cost contribution analysis is provided in Figure 8. With reference to Figure 7, the upper three subplots (average, January, and July) present the CDR potential for geographical locations in close proximity to waste heat sources, since this configuration is constrained by the availability of industrial waste heat. Thus, the maximum CDR potential is limited by the regional amount of waste heat and can result in values larger than 10 Mt/year; note the logarithmic scale of the color bar of the upper subplots. Similarly to Figure 3, the bottom three subplots present the CDR costs for the geographical regions with sufficient waste heat available.

Figure 8 is a bar plot illustrating a contribution analysis of the national averaged CDR costs for the European countries considered (on the *x*-axis). The *y*-axis shows the total CDR costs up to 1200 euro/tCO_2_ removed. The size and colors of the bar segments represent the contributions of specific processes to the total net costs of the CDR.

The highest CDR potential from DACCS can be found in areas with large availability of waste heat sources (more than 100 °C), mainly in the North-West of Europe (Belgium, The Netherlands, and the North-West of Germany) owing to extensive industrial activities, such as production of chemicals, iron and steel, nonferrous metals, nonmetallic minerals, paper and printing, and refineries. Generally, CDR costs are lower compared to those obtained under the first energy supply scenario with a decrease of average CDR costs from approximately 830 to 650 € t CO_2_^–1^, mainly due to cheaper and cleaner energy from waste heat compared to grid electricity.

**Figure 9:**
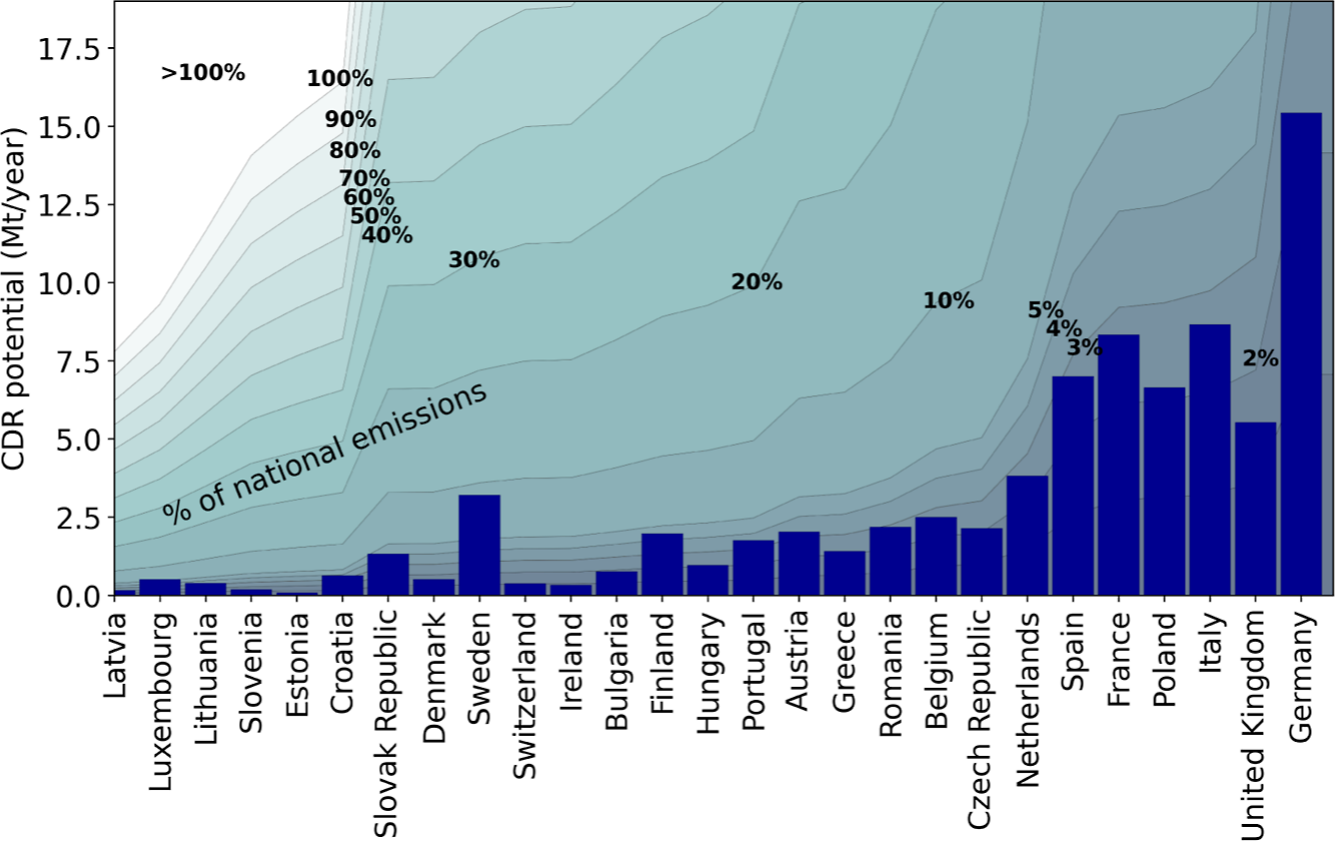
Country-specific CDR potential (Mt CO_2_/year, blue-colored bars) and the country-specific emissions (the year 2019) that can be removed with the waste heat coupled DACCS configuration (in %, isolines).

In an attempt to put these regional CDR estimates into perspective considering current waste-heat availability, Figure 9 illustrates country-specific CDR potentials on the *y*-axis with dark blue-colored bars for the countries considered on the *x*-axis. The percentages and gray areas within the figure correspond to the specific share (in percentage) of country-specific CO_2_-emissions that might be compensated for with DACCS using this energy supply scenario. For example, the total amount of annual GHG emissions in Germany is around 710 Mt CO_2_/year. The CDR potential of waste heat coupled DACCS configurations is around 16 Mt CO_2_/year; hence, the optimal deployment of this DACCS configuration can compensate for approximately 2% of the national GHG emissions in Germany.

If deployed in an optimal way and by applying current waste heat potential, DACCS can remove 3–5% of country-specific CO_2_-emissions for most European countries. Sweden stands out, as it might compensate almost 10% of its national CO_2_-emissions with a large-scale implementation of waste-heat-powered DACCS systems.

### 3.3 DAC Powered by Curtailed Renewable Energy and HTHP

This scenario assesses the potential of using curtailed electricity from renewable energy sources as an energy supply option for CO_2_ capture.^49,50^ Curtailed renewable energy is similar to waste heat in the way that they are usually both nondeployed energy sources. Figure 6b provides an overview of the installed renewable energy plants in Europe nowadays.^51^ The total European renewable-energy generation is estimated to be about 680 TW h_el_/y. Here, we focus on country-specific CDR potentials, since a geospatial map would have been a function of Figure 6b with limited overall CDR potentials.

**Figure 10:**
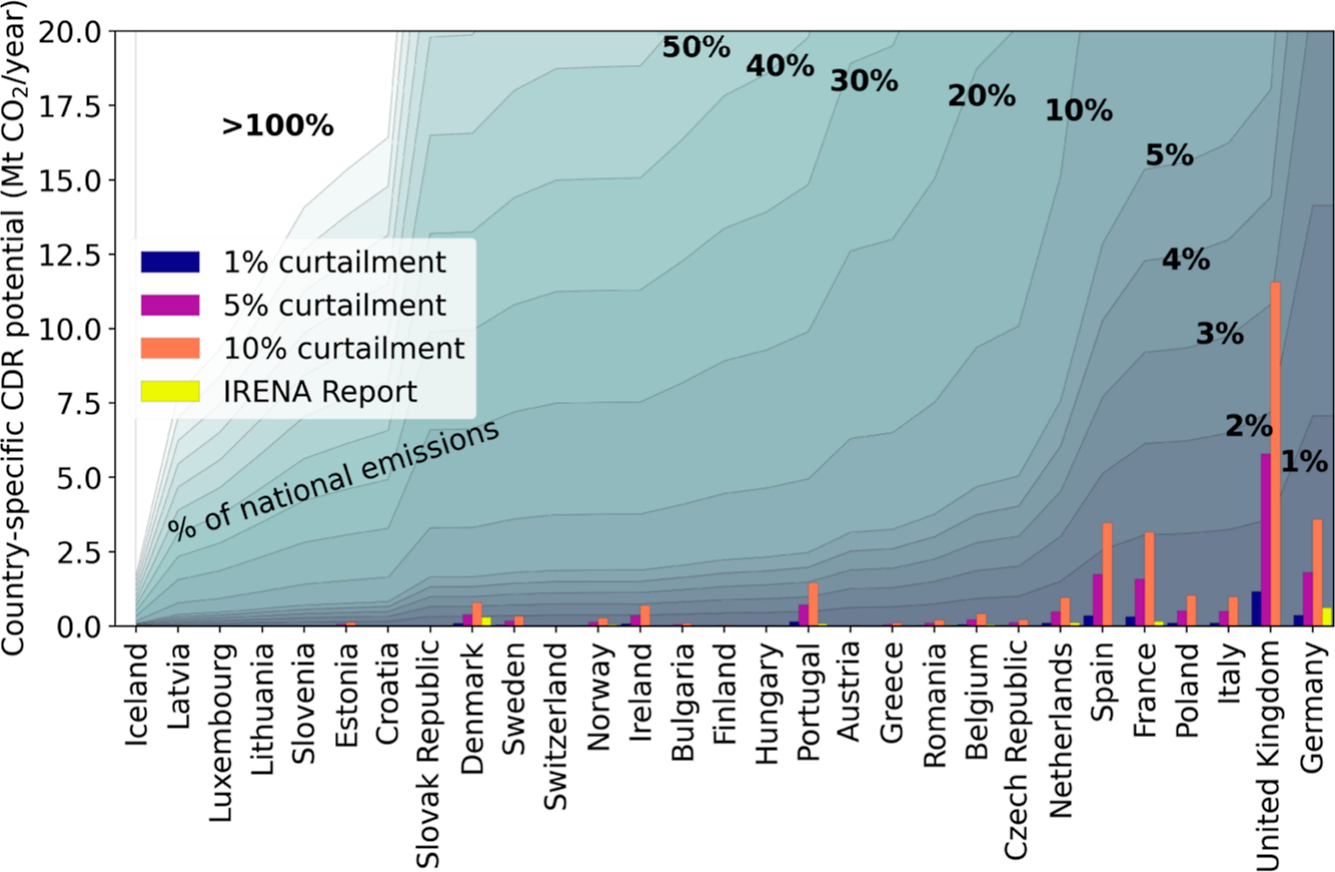
Curtailment scenarios: 1, 5, 10%, and IRENA (reMAP).

The CDR potential associated with DACCS systems powered by renewable energy was estimated for different levels of curtailment. Figure 10 shows the CDR potential as a percentage of the country-specific national CO_2_ emissions for various levels of curtailment of the renewable energy capacity currently installed, i.e., reMAP scenario from IRENA (projection of 2030), 1, 5, and 10%.^52^

Although the CDR potential via DACCS, using curtailed renewable electricity, is highly dependent on the curtailment scenario, as expected, this potential is found to be limited. The curtailment level provided by IRENA in their reMAP 2030 scenario mostly corresponds to approximately 0.5%. Under this assumption, the CDR potential would be significantly reduced (total slightly above 1.8 Mt CO_2_/y). Similarly, the IRENA scenario would limit the European DACCS removal potential to slightly less than 1.8 Mt CO_2_/y; this is too small to contribute to the deployment of DACCS at a meaningful scale.

In contrast, in a scenario with a high penetration of renewables in combination with insufficient electricity storage capacity, the curtailment regime could increase to approximately 5–10%, which would allow for a larger CDR potential in Europe (up to a total of ca. 36 Mt CO_2_/y). With higher curtailment levels, more countries might benefit from DACCS deployment using curtailed renewable energy, for example, the United Kingdom.

However, the large-scale integration of (curtailed) renewables requires measures to comply with the intermittency of renewable electricity generation, such as energy storage capacity, expansion of the power grid network, or larger DAC plants to achieve the annual CO_2_ capture rate. These measures inevitably lead to additional costs and environmental burdens that must be quantified.^8,15^ Thus, the techno-economic and environmental evaluation of such novel DACCS configurations, such as off-grid DACCS systems, is an important pointer for future work.

### 3.4 Sensitivity Analysis: the Most Influential Parameters

**Figure 11:**
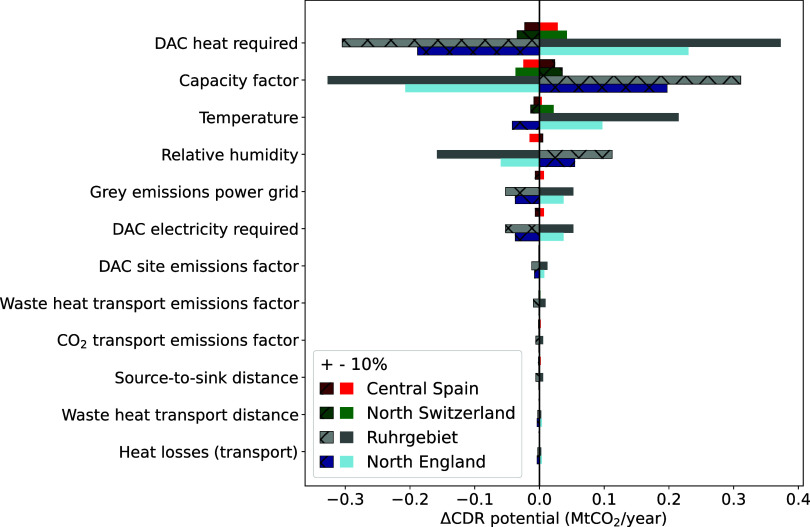
Sensitivity analysis for a set of techno-economic parameters on the CDR potential at several European locations. It is worth noting that the relative increase of the capacity factor taken is 9.5% since its maximum increase is limited to 8760 annual full load hours (compared to the 8000 h assumed in the main analysis).

Figure 11 is a sensitivity analysis for a set of techno-economic parameters and their influence on the annual CDR potential for the grid-coupled waste heat configuration. The set of parameters (on the *y*-axis) is varied by +10% and −10% for each potential DACCS deployment in four European regions; central Spain, north Switzerland, Ruhr area (Germany), and northern England. The absolute changes are presented in terms of the CDR potential on the *x*-axis.

The influence of energy requirements (mainly heat), annual load hours of the DAC plant (or capacity factor), temperature, and relative humidity on the CDR potential is significant. Increasing the annual full load hours allows for a significant reduction of capital expenditures and fixed OPEX per unit of CDR. In addition, as discussed, the local climate has a considerable influence on the energy requirements needed for CO_2_ capture.

**Figure 12:**
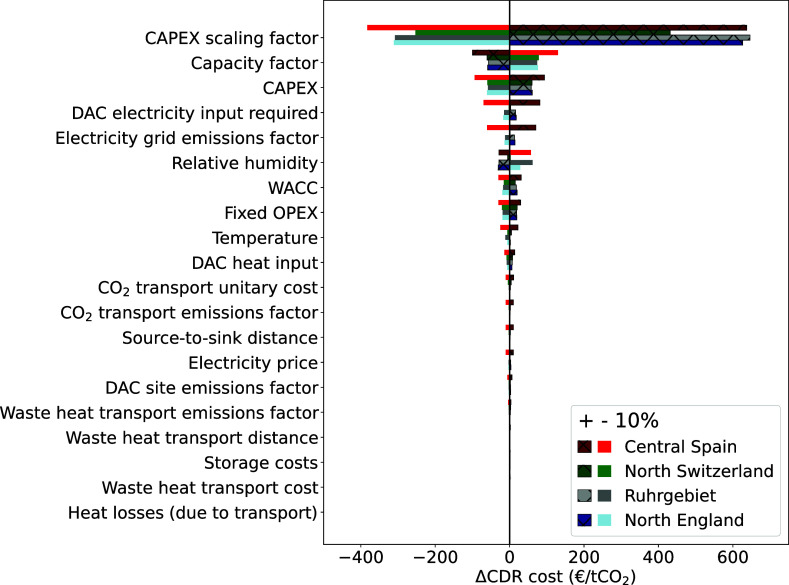
Sensitivity analysis of a set of techno-economic parameters on the CDR costs at several European locations. It is worth noting that the relative increase of the capacity factor taken is 9.5% since its maximum increase is limited to 8760 annual full load hours (compared to the 8000 h assumed in the main analysis).

In addition, Figure 12 shows a sensitivity analysis on a set of techno-economic parameters. The set of parameters (on the *y*-axis) is varied by +10% and −10% for each potential DACCS deployment at four locations; central Spain, north Switzerland, Ruhr area (Germany), and northern England. The changes are presented in terms of the absolute change of CDR costs on the *x*-axis.

The latter figure demonstrates the strong influence of the scaling factor applied on the DAC plant to account for economies of scale, annual full load hours, CAPEX, and energy requirements, respectively. Today, capital expenditure is the most influential parameter for DACCS, which might be reduced by building larger DAC plants or by achieving higher annual full-load hours.

### 3.5 Limitations, Solutions, and Future Work

The analysis presented in this paper is valuable in providing general guidelines and insights on the optimal location and roll-out of DACCS systems, but it is important to acknowledge that it is based on certain simplifications and assumptions. While these simplifications were necessary in order to conduct the current analysis, it is crucial to recognize that they may not hold in all cases. Further studies should aim to address these assumptions and explore their potential impacts on the results. However, this should not diminish the usefulness of the current work, which serves as a valuable starting point for future investigations. In the following, we address the main limitations of our analysis and suggest potential ways forward to cope with them in future investigations.

First, environmental trade-offs are not considered in detail since we focus on costs and quantify GHG emissions from different DACCS configurations in Europe. However, the assessment of the environmental performance of DACCS should consider additional environmental burdens, as well. Thus, a potential large-scale deployment of DACCS requires an evaluation of environmental trade-offs using a life cycle assessment approach.^15^ For example, our analysis excludes the (life cycle) land footprint of DAC plants or the available space in the locations under consideration. This is an important factor to consider, as the land requirements of DAC plants can vary significantly depending on their size and design.^8^ For example, our analysis excludes the life-cycle land footprint of DAC plants or land availability in the locations considered. In fact, land competition with agriculture and building might be an issue. For example, centralized large-scale DAC plants, such as those planned by Climeworks,^53^ may require a larger land area compared to decentralized DAC concepts, which envision the use of multiple smaller plants as proposed by Noya and NeoCarbon.^19,20^ Although the availability of suitable land may not be an issue for all locations and DAC concepts,^8^ it is valuable to assess the land requirements and availability for any DAC project to ensure its feasibility and sustainability. Finally, public acceptance could be another factor hindering the large-scale deployment of DACCS systems, especially in regions with a high population density.^54^

Our analysis shows that the CDR costs of DACCS are substantially lower when low-carbon energy sources (i.e., decarbonized grid electricity) are employed for CO_2_ capture. Previous findings^7,8^ confirm that CDR efficiencies of DACCS coupled to low-carbon energy sources, such as solar PV and waste heat, are around 80–95%, resulting in a minor increase of net CDR costs by 5–25%. In contrast, using carbon-intensive energy sources, assuming, for example, a CDR efficiency of 50%, would increase net CDR cost by a factor 2 (i.e., 200%), which shows the importance of considering life cycle CDR efficiencies and reducing emissions over the entire supply chain of any CDR technology. Further, our analysis applies country-specific average GHG emission factors for the electricity grid network. Alternatively, a consequential approach using marginal GHG emission factors might be applied since the implementation of grid-coupled DACCS systems requires an expansion of the electricity generators coupled to the grid electricity network. In Europe, additional grid electricity capacity is, to a large extent, delivered by renewables nowadays.^55^ Consequently, GHG emission factors of marginal electricity generators are mainly based on renewables. Applying marginal GHG emission factors could significantly reduce GHG emissions from the considered DACCS systems, especially those relying on grid electricity. Thus, our approach can be perceived as conservative, and a larger CDR potential through DACCS might be delivered by applying marginal GHG emission factors. Especially for grid-coupled DACCS systems, it is crucial to install additional grid capacity based on renewables to fully decarbonize the grid electricity network in order to prevent the generation and absorption of GHG-intensive grid electricity.^8^

Our cost quantification is based on the current cost figures of DACCS, and hence, we need to pay more attention to potential cost reductions that may occur in the future due to technology learning. On the one hand, it is possible that future technological advancements could lead to reduced costs for DACCS systems. On the other hand, it is important to note that there is a high level of uncertainty surrounding these potential cost reductions,^28,47^ and it is difficult to predict the extent and timing of such reductions accurately. Prospective cost analyses and environmental LCA, which we want to address in future work, can be helpful in exploring future costs and the environmental performance of DACCS. Despite this limitation, the general trends and conclusions drawn in our study would still be valid, especially in providing a comparative assessment among different locations and energy supply options.

Our results indicate that climate-dependent factors (e.g., ambient temperature and humidity) have substantial influence on CDR potential and cost. Additional location-specific factors could be included in future spatially explicit techno-economic analyses. For example, more frequent sorbent-replacement might be needed for DAC units installed in unsuitable climates,^56^ which would further increase CDR costs and reduce the CDR potential.

Additionally, our analysis focuses on one DAC technology, namely, solid sorbent DAC, as currently exploited in Climeworks’ technology and also proposed by other start-ups in the field. Therefore, our results cannot be generalized to other DAC technologies that are not based on solid sorbents, as the dependence of performance on climate and weather is very specific to the DAC technology analyzed.

Here, we consider only CO_2_ transport via pipelines and its associated cost; transport distances are assumed to be corresponding to straight-line distances (i.e., “as the crow flies”) since we consider large-scale DAC facilities in the main analysis (1 Mt CO_2_/a or larger). Due to uncertainties regarding the topology of pipeline networks, we choose to disregard the inclusion of a tortuosity factor in the analysis, as its impact on transport costs is assumed to be considered in the uncertainty of pipeline costs. However, pipeline transport networks are not developed yet, and recent research^35^ demonstrates that container-based transport (i.e., relying on CO_2_ loaded onto a container that is transported via truck, railway, and ship/barge) exhibit substantially higher emissions and costs per unit of distance of CO_2_ transported. For example, the most economic transport solution available in the short term to deliver CO_2_ captured at a location in inland Europe (i.e., Switzerland) to a storage site located in the North Sea (i.e., Northern Lights in Norway) consists of a combination of pipeline, truck, train, ship, and truck transport resulting in a cost of 160 € t CO_2_^–1^.^57^ For the same source-to-storage connection based on straight-distance pipelines, this analysis assumes a transport cost of ca. 60 € t CO_2_^–1^, almost three times smaller than the current multimodal cost estimate. Thus, future geospatial analysis should also account for current transport modes of CO_2_, as pipeline transport networks will be available only in a long-term time horizon. In addition, the costs for storage and transport of CO_2_ exclude cost penalties between the country of the CO_2_ source and the CO_2_ sink due to uncertainties related to such costs. Future assessments should consider two additional aspects; (i) legal and socio-economic aspects of sequestration prioritization and (ii) social acceptance of CO_2_ capture and storage since the acceptance for storing CO_2_ from other countries might be lower, in particular, within the country of the CO_2_ sink.^58,59^

Finally, our results reveal considerable CDR potentials from DACCS powered by currently available waste-heat sources in the northwest of Europe, especially in Belgium, The Netherlands, and the Ruhr area in Germany, mainly owing to extensive industrial activities. Nevertheless, the availability of waste-heat sources will likely be reduced in the near future, due to the phase-out of conventional power plants and the implementation of low-carbon energy carriers.^8^

Despite the identified limitations, our paper still offers a valuable initial assessment, providing a foundation for further studies in the field.

## 4 Implications for DACCS Deployment

This work aims to determine the optimal geospatial distribution, CDR potential, and costs of a potential large-scale DACCS deployment in Europe. Different DACCS configurations are considered: (1) DAC powered by electricity from the grid and heat supplied by a high-temperature heat pump, (2) DAC powered by electricity from the grid and heat supplied locally by available waste-heat sources, and (3) DAC powered by electricity from curtailed renewable sources and heat supplied by a high-temperature heat pump operated with curtailed renewable electricity.

Our findings reveal that there are several factors that drive the CDR potential and cost of grid-connected DACCS systems, mainly the price and GHG intensity of grid electricity, the CO_2_ transport distance to the nearest CO_2_ storage location, the location-specific climate conditions, and the availability of energy sources. Additionally, the country-specific interest rate, and thus the upfront investment, is found to be an important factor influencing the CDR costs. These findings have different implications for enabling smooth large-scale deployment of DACCS in Europe.

First, DACCS systems require substantial energy sources for the CO_2_ capture. DACCS should, therefore, be deployed at geographical locations with vast energy sources available, for example, in close proximity to waste heat sources, (excess) renewables, and/or low-carbon grid-electricity networks. Potential suitable DACCS locations can be found in the Northwest of Europe (The Netherlands, Denmark, Sweden, and Iceland), France, and Switzerland. Second, DACCS systems require permanent storage of CO_2_ in a geological storage formation to ensure long-term CO_2_ removals. This is demonstrated by the poor performance of the evaluated DAC technology in hot climates, particularly in locations far from storage sites. However, our analysis also identifies regions in central Europe, such as South France and Switzerland, with significant CDR potentials. The CDR potential and costs of DAC plants potentially installed in these regions and based on solid sorbents (with different climate dependencies) or solvents would benefit from the establishment of an international CO_2_ transport network.

Further, DACCS systems should only be installed by using low-carbon energy sources. Therefore, one prevailing factor is the decarbonization of local electricity generation to increase the CO_2_ removal efficiency from DACCS systems. In addition, the innovation of DACCS is of crucial importance along the entire supply chain, which should result in a reduction of energy consumption for CO_2_ capture and lower capital expenditures.

Finally, our geospatial analysis reveals substantial differences in terms of CDR efficiency and costs between geographical locations. This implies that location-specific assessments are required to find suitable DACCS configurations considering the cost, environmental, and social aspects. Our work provides insights into the most suitable grid-coupled DACCS locations within the European context for the deployment of large-scale CDR in a net-zero global energy system.

## Supporting Information Available

Country-specific input parameters used and main data sources; illustration of the theoretical CO_2_ storage formations in Europe; monthly DAC energy requirements; monthly CDR costs; scaling effects capex; CDR costs as a function of DAC size for different scaling factors; and the uncertainty range of pipeline transport costs (PDF)
