# Supplementary material for: Assessment of Potential and Techno-Economic Performance of Solid Sorbent Direct Air Capture with CO2 Storage in Europe
Source: Environ Sci Technol. 2024 Jun 3;58(24):10567–81. doi: 10.1021/acs.est.3c10041 (PMC11191593; doi:10.1021/acs.est.3c10041)
Supplement: Supplementary file 1 — es3c10041_si_001.pdf [file es3c10041_si_001.pdf]

Assessment of potential and techno-economic performance of solid sorbent direct air capture with CO_2 storage in Europe

Supporting Information (SI)

Tom Terlouw[a,b,c], Daniel Pokras[a], Viola Becattini[a*], and Marco Mazzotti[a*]

[a] Separation Processes Laboratory, Institute of Energy and Process Engineering, ETH Zurich, Zurich 8092, Switzerland

[b] Chair of Energy Systems Analysis, Institute of Energy and Process Engineering, ETH Zurich, Zurich 8092, Switzerland

[c] Technology Assessment Group, Laboratory for Energy Systems Analysis, 5232 Villigen PSI, Switzerland

[*] *Corresponding authors: viola.becattini@ipe.mavt.ethz.ch & marco.mazzotti@ipe.mavt.ethz.ch*

Summary: 12 pages, 2 tables, and 6 figures.

Contents

S1 Additional Tables **S3**

S1.1 Country-specific input parameters S3

S1.2 Data Sources S3

S2 Additional Figures **S5**

S2.1 Theoretical storage formations S5

S2.2 Monthly DAC energy requirements: heat and electricity S6

S2.3 Monthly CDR costs: full grid alternative S7

S2.4 Capex scaling factors S8

S2.5 Transport cost of CO_2 . . . . S10

References S11

List of Figures

S1 Theoretical storage formations considered. S5
S2 Monthly energy requirements for DAC. S6
S3 Monthly CDR costs. S7
S4 Scaling effects capex compared to the one considered (0.91). S8
S5 CDR costs as a function of DAC size for different scaling factors. S9
S6 Uncertainty range for the costs of pipeline transport of CO_2. . . . . S10

List of Tables

S1 Country-specific input parameters used. S3
S2 Data sources S4

S1 Additional Tables

S1.1 Country-specific input parameters

Table S1 provides country-specific data used in the geospatial analysis for national emissions, electricity prices, and weighted average cost of capital (WACC).

Table S1: Country-specific input parameters used.

	**National Emissions [$MtCO_2$ $year^{-1}$]**[1]	**Electricity price [€ kWh^{-1}]**[2]	**WACC [–]**[3,4]
**Austria**	63.1	0.088	0.040
**Belgium**	93.7	0.080	0.045
**Bulgaria**	40.9	0.081	0.089
**Switzerland**	37.5	0.043	0.045
**Cyprus**	7.2	0.118	0.135
**Czech Republic**	100.9	0.072	0.065
**Germany**	707.7	0.085	0.054
**Denmark**	33.1	0.054	0.036
**Spain**	257.0	0.079	0.059
**Estonia**	15.3	0.068	0.081
**Finland**	44.6	0.063	0.050
**France**	307.0	0.085	0.040
**United Kingdom**	360.7	0.107	0.059
**Greece**	65.0	0.082	0.089
**Croatia**	16.4	0.090	0.100
**Hungary**	46.4	0.085	0.079
**Ireland**	37.7	0.127	0.077
**Iceland**	1.7	0.071	0.071
**Italy**	324.9	0.086	0.056
**Lithuania**	11.6	0.087	0.030
**Luxembourg**	9.3	0.082	0.071
**Latvia**	7.8	0.081	0.066
**Malta**	1.5	0.133	0.064
**Netherlands**	151.4	0.068	0.050
**Norway**	37.6	0.042	0.076
**Poland**	311.9	0.080	0.074
**Portugal**	49.5	0.079	0.063
**Romania**	75.2	0.089	0.074
**Slovakia**	33.0	0.098	0.052
**Slovenia**	14.1	0.081	0.060
**Sweden**	36.0	0.064	0.050

S1.2 Data Sources

Table S2 shows the main data sources used in our work.

Data Type	Description	Author(s)	Ref.
Waste Heat Sources EU	Locations, heat quantity and quality of all reported waste heat sources in the EU	Tobias Fleiter et al.	[5]
Waste Heat Sources CH	Locations, heat quantity and quality of all reported waste heat sources in Switzerland	Chambers et al.	[6]
Renewable Energy Sources	Locations and electricity capacities all renewable energy sources in Europe	Global Energy Observatory	[7]
Geological Sinks	Locations of various geological storage sites in Europe	Various Sources	[8–18]
Theoretical Storage Capacities	Locations of various geological storage sites in Europe with Theoretical Storage Capacities	Anthonsen and Christensen	[19]
European Basemap	Basemap of Europe used to overlay data analysis	Eurostat	[20]
European Electricity Grid Data	Electricity prices and CO_2 emssions per region/country	Ecoinvent Database 3.8	[21]
CO_2 Transport Costs Data	Unit cost of CO_2 transport for various transport methods	Oeuvray et al.	[22]
Optimal DAC Performance Data	Model correlating inlet conditions with DAC energy requirements	Wiegner et al.	[23]
Elevation Data	Altitude elevation throughout the world	Global Solar Atlas	[24]

Table S2: Data sources

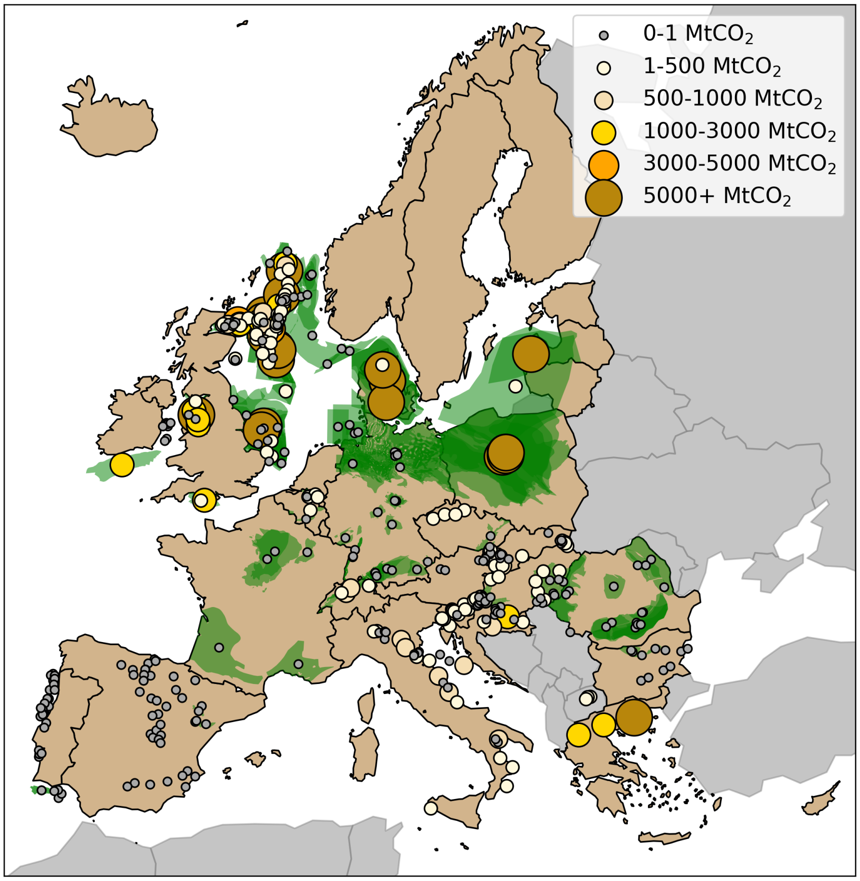

Figure S1: Theoretical storage formations considered.

S2 Additional Figures

S2.1 Theoretical storage formations

Figure S1 shows the theoretical storage sites available in Europe. It is worth noting that only theoretical storage sites larger than 5 Gt CO_2 are included in the main analysis since we are interested in the large-scale deployment of DACCS.

S2.2 Monthly DAC energy requirements: heat and electricity

Figure S2 presents the DAC energy requirements (heat and electricity) for the selected sorbent for each month in Europe.

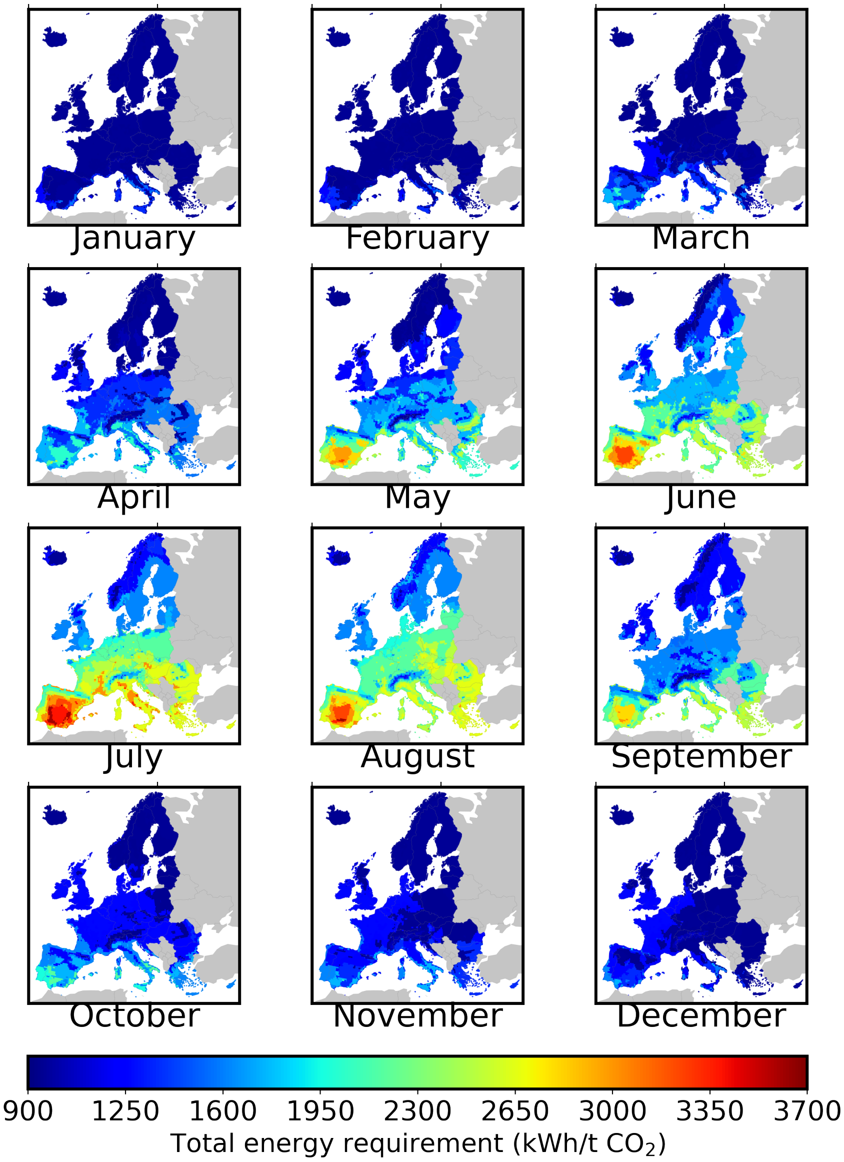

Figure S2: Monthly energy requirements for DAC.

S2.3 Monthly CDR costs: full grid alternative

Figure S3 presents the monthly CDR costs when using a specific annual month for the entire cost analysis in Europe.

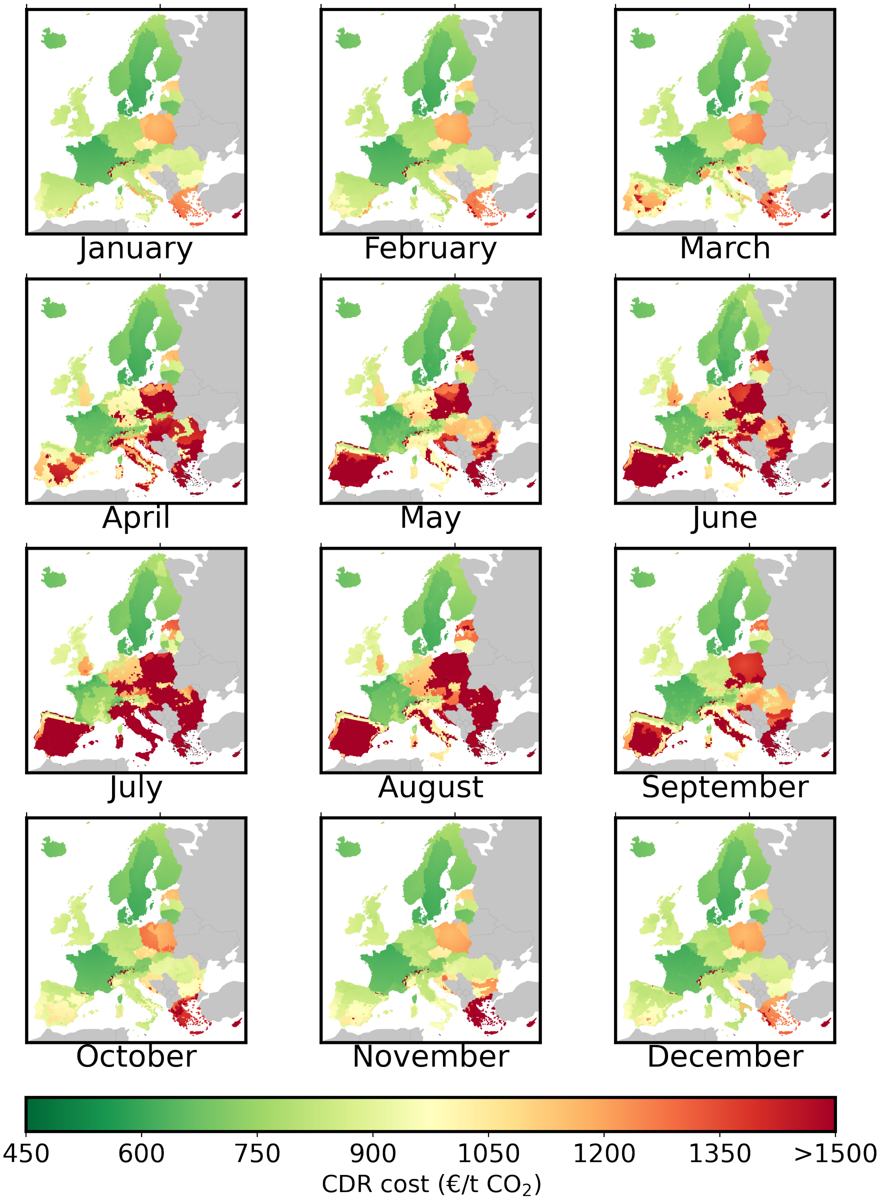

Figure S3: Monthly CDR costs.

S2.4 Capex scaling factors

Different capex scaling factors are provided in Figures S4–S5. Here, we compare our approach on economies of scale (using a scale factor of 0.91) and compare it with the learning and economies of scale correlations of Young et al.[25]. These figures illustrate that our DACCS cost outcomes fall within the range of their work.

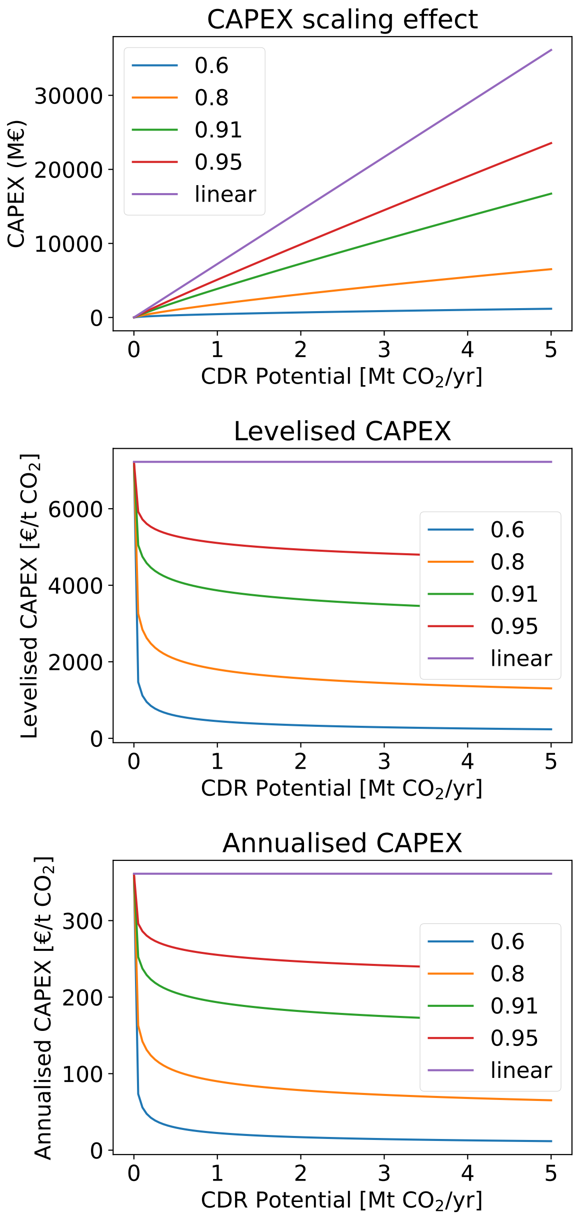

Figure S4: Scaling effects capex compared to the one considered (0.91).

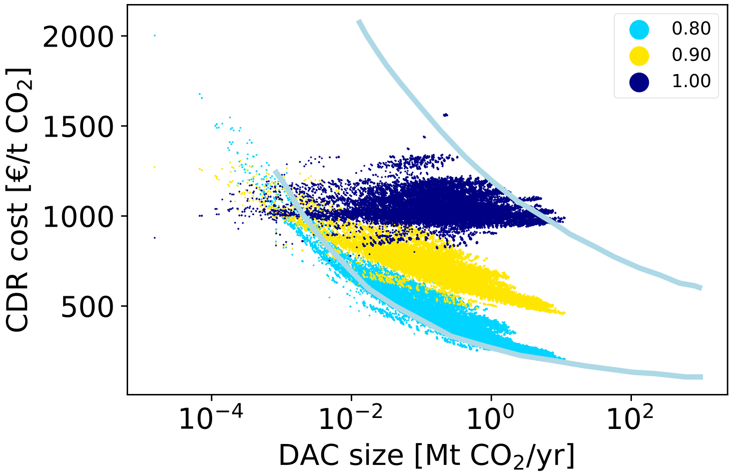

Figure S5: CDR costs as a function of DAC size for different scaling factors.

S2.5 Transport cost of CO_2

Figure S6 shows the uncertainty range in terms of costs of transportation of CO_2. The low unit cost scenario refers to onshore pipeline transport with distances of 1,000 kilometer and with a capacity of 10 $MtCO_2/a$ while the high unit cost refers to onshore pipeline transport with distances of 1,000 kilometer and with a capacity of 0.1 $MtCO_2/a$. The chosen average scenario, in the main analysis, refers to 1$MtCO_2/a$ capacity.

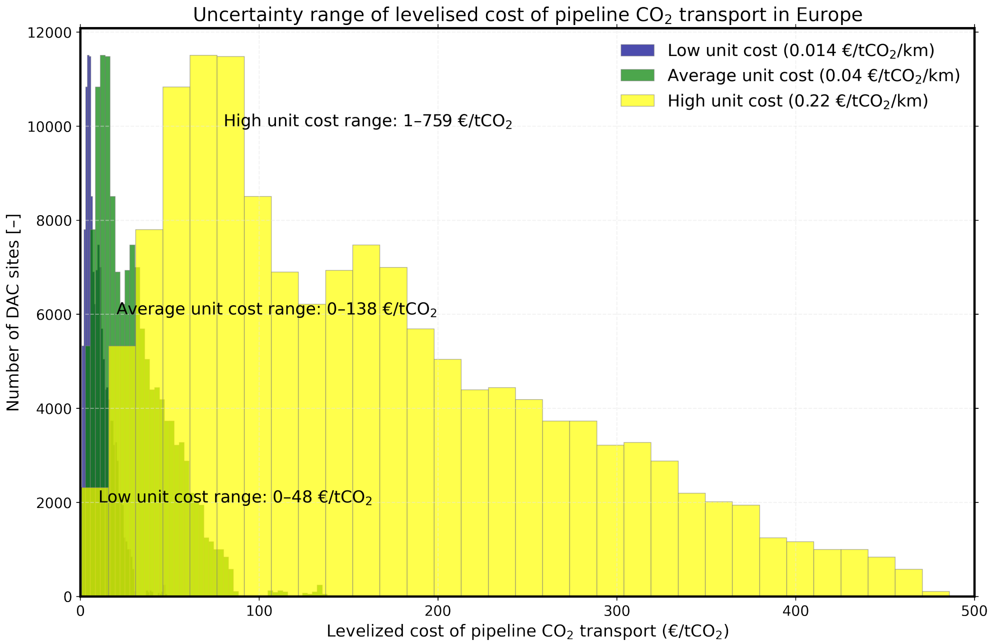

Figure S6: Uncertainty range for the costs of pipeline transport of CO_2.

References

1. *Statistics - Eurostat* Date Last Accessed April 12, 2022. 2019. https://ec.europa.eu/eurostat/databrowser/view/sdg_13_10/default/table?lang=en.
2. Eurostat. *Electricity prices for non-household consumers - bi-annual data (from 2007 onwards)* Date Last Accessed August 30, 2022. https://ec.europa.eu/eurostat/databrowser/view/nrg_pc_205/default/table?lang=en.
3. Performance Review Body of the Single European Sky. Study on Cost of Capital Methodology review. Date Last Accessed August 30, 2022. https://eu-single-sky.transport.ec.europa.eu/document/download/e2ec2658-d099-4fcd-af77-c348672fae95_en?filename=Cost%20of%20capital%20methodology%20review_0.pdf&prefLang=ro (2019).
4. *BEREC Report Regulatory Accounting in Practice 2019 (including WACC chapter)* Date Last Accessed August 30, 2022. 2019. https://berec.europa.eu/eng/document_register/subject_matter/berec/reports/8907-berec-report-regulatory-accounting-in-practice-2019-including-wacc-chapter.
5. Tobias Fleiter (Fraunhofer ISI) *et al. Quantification of Synergies between Energy Efficiency First Principle and Renewable Energy Systems: D5.1 Excess heat potentials of industrial sites in Europe Documentation on excess heat potentials of industrial sites including open data file with selected potentials* tech. rep. Date Last Accessed August 30, 2022 (Aalborg University, Aalborg, 2019). https://seenergies.eu/https://s-eenergies-open-data-euf.hub.arcgis.com/datasets/a6a1e8e95514413a90bbb2e40515fdb2_0/explore?location=46.455944\%2C31.806195\%2C4.52.
6. Zuberi, M. J. S. *et al.* Excess heat recovery: An invisible energy resource for the Swiss industry sector. *Applied Energy* **228,** 390–408 (2018).
7. Global Energy Observatory, Google, KTH Royal Institute of Technology in Stockholm, Enipedia & World Resources Institute. *Global Power Plants Database* Date Last Accessed August 30, 2022. 2018. https://datasets.wri.org/dataset/globalpowerplantdatabase.
8. *Ravenna CCS - The CCUS Hub* Date Last Accessed August 30, 2022. 2022. https://ccushub.ogci.com/focus_hubs/ravenna/.
9. *Liverpool Bay: biodiversity and environmental safety in the UK — Eni* Date Last Accessed August 30, 2022. 2022. https://www.eni.com/en-IT/operations/united-kingdom-liverpool-bay.html.
10. *ATHOS Project Details* Date Last Accessed August 30, 2022. 2022. https://www.geos.ed.ac.uk/sccs/project-info/2507.

11. *Home - Zero Carbon Humber* Date Last Accessed August 30, 2022. 2022. https://www.zerocarbonhumber.co.uk/.

12. *Net Zero Teesside — The UK's first decarbonised industrial cluster* Date Last Accessed August 30, 2022. 2022. https://www.netzeroteesside.co.uk/.

13. *The Acorn Project* Date Last Accessed August 30, 2022. 2022. https://theacornproject.uk/.

14. *Project - Porthos* Date Last Accessed August 30, 2022. 2022. https://www.porthosco2.nl/en/project/.

15. *Project Greensand — CO2 Lagring* Date Last Accessed August 30, 2022. 2022. https://www.projectgreensand.com/.

16. *Northern Lights* Date Last Accessed August 30, 2022. 2022. https://norlights.com/.

17. *Caledonia Clean Energy Project Details* Date Last Accessed August 30, 2022. 2022. https://www.geos.ed.ac.uk/sccs/project-info/98.

18. Geothermal, H. Geothermal Sustainability Assessment Protocol. Date Last Accessed August 30, 2022. https://arsskyrsla2021.or.is/documents/294/Hellisheidi_-_Geothermal_Sustainability_Assessment_Protocol_-_Final_Report_22062018.pdf (2018).

19. Lyng Anthonsen, K. & Peter Christensen, N. *EU Geological CO2 storage summary* Date Last Accessed August 30, 2022. 2021. https://cdn.catf.us/wp-content/uploads/2021/10/20183953/EU-CO2-storage-summary_GEUS-report-2021-34_Oct2021.pdf.

20. eurostat. *NUTS Maps - NUTS - Nomenclature of territorial units for statistics - Eurostat* Date Last Accessed August 30, 2022. https://ec.europa.eu/eurostat/web/nuts/nuts-maps.

21. ecoinvent. *ecoinvent 3.8* Date Last Accessed November 3, 2022. 2022. https://ecoinvent.org/ecoinvent-38-release/.

22. Oeuvray, P., Burger, J., Roussanaly, S., Mazzotti, M. & Becattini, V. Multi-criteria assessment of inland and offshore carbon dioxide transport options. *Journal of Cleaner Production* **443,** 140781 (2024).

23. Wiegner, J. F., Grimm, A., Weimann, L. & Gazzani, M. Optimal Design and Operation of Solid Sorbent Direct Air Capture Processes at Varying Ambient Conditions. *Industrial & Engineering Chemistry Research* **61,** 12649–12667 (2022).

24. Global Solar Atlas 2.0. *Global Solar Atlas 2.0* Date Last Accessed Thursday, January 13, 2022. 2022. https://globalsolaratlas.info.

25. Young, J. *et al.* The cost of direct air capture and storage can be reduced via strategic deployment but is unlikely to fall below stated cost targets. *One Earth* **6,** 899–917 (2023).
